# Is It Time to Rethink the Relationship Between Subscapularis Tears and the Long Head of Biceps Tendon?

**DOI:** 10.7759/cureus.54139

**Published:** 2024-02-13

**Authors:** Rohan Prakash, Andrew P Dekker, Chetan Modi, Thomas Lawrence, Stephen Drew, Gevdeep Bhabra

**Affiliations:** 1 Trauma and Orthopaedics, University Hospitals Coventry and Warwickshire, Coventry, GBR

**Keywords:** ultrasound, subluxation, tear, biceps, subscapularis

## Abstract

Introduction

Pre-operative diagnosis of subscapularis tears remains a difficult challenge. Ultrasound has been shown to be ineffective at directly detecting subscapularis tears. It has been widely accepted that medial subluxation of the long head of biceps tendon (LHBT) is associated with full-thickness subscapularis tears. The aims of this study are to assess whether LHBT subluxation on ultrasound scanning has any predictive value for subscapularis tears and to determine the relationship between LHBT subluxation and subscapularis tears at arthroscopy.

Methods

Pre-operative ultrasound and arthroscopic findings for patients undergoing arthroscopic rotator cuff repair at our institution between March 2011 and January 2016 were analysed. The accuracy of LHBT subluxation on ultrasound and at arthroscopy as a predictor of subscapularis tears at arthroscopy was calculated. The correlation between LHBT subluxation and subscapularis tears was determined. A standardised technique was used for ultrasound scans, and the grade of the sonographer was recorded.

Results

Three hundred fifty-nine rotator cuff repairs were performed. Twenty-four patients had a subluxed LHBT. Ultrasound was poorly sensitive (50%), and a subluxed LHBT on ultrasound only correlated very weakly with subscapularis tears at arthroscopy (R = 0.268, p<0.001). At arthroscopy, 92 patients had full-thickness subscapularis tears. Of these, only 16 patients (17%) had a subluxed/dislocated LHBT. Of the 24 patients with a subluxed LHBT, eight had no subscapularis tears. Thus, LHBT subluxation/dislocation only correlated weakly with full-thickness subscapularis tears (R=0.252, p<0.001).

Conclusion

Due to their close anatomical relationship, traditional teaching suggests subscapularis tears are associated with medial LHBT subluxation. Our data indicate that, contrary to popular belief, the two are only weakly correlated. In our series, the majority of patients with subscapularis tears (83%) had their LHBT in-groove. The authors therefore recommend high vigilance during arthroscopy for the diagnosis and repair of subscapularis tears, regardless of pre-operative ultrasound findings and the intra-operative position of the LHBT.

## Introduction

The subscapularis is the largest and strongest of the rotator cuff muscles, with cadaveric studies suggesting it contributes more than 50% of rotator cuff strength [1]. It is crucial to normal shoulder biomechanics, providing an anterior balance to the transverse and coronal force couples [2].

Subscapularis tendon tears may be degenerate in origin or result from trauma [3]. Patients with subscapularis tears may present with anterior shoulder pain, along with pain and weakness in shoulder elevation or internal rotation. However, they can be difficult to diagnose with clinical examination. Barth et al. compared four common clinical tests for subscapularis tears with arthroscopic findings and found the bear-hug test to be the most sensitive; however, even this test was found to be only 60% sensitive for identifying subscapularis tears [4].

Historically, subscapularis tears have been difficult to identify intra-operatively with open surgery, but the advent of arthroscopy has meant that they are more readily recognised and repaired. The pre-operative diagnosis of subscapularis tears, however, remains a difficult challenge.

Ultrasound has been proven to be effective in identifying superior and posterior rotator cuff tears [5,6]. However, the sensitivity for subscapularis tears was questioned by Ward et al. (2018), who studied 286 patients undergoing shoulder arthroscopy and found that subscapularis tear ultrasound was very poorly sensitive (13%). Magnetic resonance imaging (MRI) was only marginally more sensitive (30%), although specificity was high [7]. Similarly, we have found that in our institution, ultrasound is highly sensitive for diagnosing supraspinatus tears [8] but not subscapularis tears (unpublished results). There is a long-held view that there is a close relationship between subscapularis tears and the long head of biceps tendon (LHBT) and that medial subluxation of the LHBT is associated with full-thickness subscapularis tears [9]. This is due to the contribution of the subscapularis tendon to the medial border of the biceps sling or pulley [10], which is also contributed to by the superior glenohumeral ligament (SGHL) and coracohumeral ligament (CHL). However, the relative contributions of the different components to the LHBT stability have been a topic of debate more recently [11]. Arthroscopic and histoanatomical studies have suggested the LHBT can sublux medially without subscapularis tears and that the SGHL/CHL complex provides greater stability to the LHBT compared to the subscapularis tendon [11,12].

Recent evidence has also questioned the value of long-head biceps subluxation on MRI in predicting subscapularis tears [13,14]. Shi et al. (2015) concluded that if the LHBT was not subluxed, a subscapularis tear was very unlikely (negative predictive value 97%) [14]. However, only 35% of patients who had a subluxed LHBT on an MRI scan were found to have a subscapularis tear at arthroscopy. The predictive value of LHBT subluxation or dislocation on ultrasound for subscapularis tears has yet to be definitively established. However, Fujiwara et al. (2022) suggest the position of LHBT on pre-operative ultrasound can be used to predict the presence of intra-operative subscapularis tears [15]. As a dynamic imaging modality, we suppose that ultrasound may be more useful than static imaging, as recently illustrated by Zhu et al. (2022), who found ultrasound to be superior to MRI in diagnosing partial-thickness subscapularis tears [16].

The primary aim of this study is to assess the relationship between LHBT subluxation and subscapularis tears at arthroscopy. The secondary aim is to investigate further whether LHBT subluxation on ultrasound scanning has any predictive value as a surrogate marker for subscapularis tears.

This article was previously presented as a poster presentation at the 2022 London Shoulder Meeting on May 20th, 2022. 

## Materials and methods

In this retrospective cohort study, analysis of pre-operative ultrasound scans and arthroscopic findings was performed for all patients undergoing arthroscopic rotator cuff repair at our institution between March 2011 and January 2016. Those without pre-operative ultrasound scans and those who had an MRI rather than an ultrasound scan were excluded. Revisions or repeat procedures were also excluded.

Subscapularis arthroscopic findings were divided into the following categories based on the Yoo and Rhee classification [17]: normal, tendinopathic (type 1), partial thickness tear (type 2A), full-thickness upper border tear (type 2B), and full thickness complete tear (types 3-5).

LHBT position on ultrasound was classified as 'in-groove', subluxed, or dislocated. Arthroscopic LHBT findings were classified as in-groove (normal), in-groove (frayed and tendinopathic), subluxed, dislocated, or ruptured. For each patient, the pre-operative ultrasound report and intra-operative notes were reviewed, with extracted data recorded in a spreadsheet.

The accuracy of LHBT subluxation or dislocation, both on ultrasound and at arthroscopy, as a predictor of subscapularis tears at arthroscopy was calculated using 95% confidence intervals. The correlation between LHBT subluxation/dislocation and subscapularis tears was calculated using Spearman’s rank correlation coefficient and Chi-squared tests. Statistical analysis was carried out using the SPSS® Statistics software platform (IBM Corp. Released 2021. IBM SPSS Statistics for Windows, Version 28.0. Armonk, NY: IBM Corp).

The grade of the person performing the ultrasound was recorded (consultant musculoskeletal radiologist or sonographer) and accounted for in the statistical analysis. All ultrasound scans were performed using the Logiq E9 GE machine (GE HealthCare, Chicago, USA) using a high-resolution linear probe 9-15 MHz.

A standardised technique was used for all ultrasound scans. The subscapularis was assessed with the patient seated, initially with their arm in a neutral position resting on the patient’s lap, and then with the patient externally rotating their arm with their elbow kept close to their side. The transducer was first placed in a transverse orientation and then in a longitudinal orientation to also allow visualisation of the subscapularis in the short-axis view. This technique enables dynamic assessment of the subscapularis tendon and LHBT in its bicipital groove.

## Results

Three hundred fifty-nine rotator cuff repairs were performed on 207 male and 152 female patients. The median age was 61 years (interquartile range 54-67). One hundred seventy-five (49%) patients had a normal subscapularis at arthroscopy. The subscapularis was tendinopathic in 51 (14%) patients, had a partial thickness tear (<50% depth) in 41 (11%) patients, had an upper border full thickness tear in 54 (15%) patients, and had a full thickness retracted tear in 38 (10%) patients (Table 1).

**Table 1:** Subscapularis tendon condition at arthroscopy

Subscapularis tendon condition	Number of patients (n)
Normal	175
Tendinopathic	51
Partial thickness tear	41
Upper border tear (full thickness)	54
Full-thickness retracted tear	38
Total	359

At arthroscopy, of the 92 patients with subscapularis tears, 16 patients (17%) had a subluxed/dislocated LHBT; 76 out of the 92 patients (82.6%) had their LHBT in-groove. Conversely, in the 267 patients with no full-thickness subscapularis tear, the LHBT was in-groove in 259 cases (97%) and subluxed/dislocated in eight cases. Twenty-four patients had a subluxed LHBT, and of these eight, none had a subscapularis tear. Thus, LHBT subluxation and dislocation only correlated weakly with full-thickness (including upper border) subscapularis tears (R=0.252, p<0.001; Table 2).

**Table 2:** Relationship between LHBT subluxation/dislocation on ultrasound and arthroscopy with subscapularis tears

	Ultrasound LHBT subluxed/dislocated vs. arthroscopic subscapularis full thickness tear (including upper border)	Arthroscopy LHBT subluxed/dislocated vs arthroscopic subscapularis full thickness tear (including upper border)
Actual tears	92 patients	92 patients
Normal	267 patients	267 patients
True positive	21 patients	16 patients
False positive	13 patients	8 patients
False negative	71 patients	76 patients
True negative	254 patients	259 patients
Sensitivity	22.8%	17.4%
Specificity	95.1%	97.0%
Positive predictive value	61.8%	66.7%
Negative predictive value	78.2%	77.3%
Accuracy	76.6%	76.6%
P (Chi2)	<0.001	<0.001
R (Spearman)	0.268	0.252

In our study, a subluxed or dislocated LHBT on ultrasound only correlated very weakly with subscapularis tears (R=0.268, p<0.001; Table 2). Of the 92 patients with arthroscopically confirmed subscapularis tears, 21 were found to have a subluxed LHBT on ultrasound (sensitivity of 22.8%). Thirty-four patients were diagnosed with a subluxed LHBT on ultrasound; however, in this group, only 21 were found to have subscapularis tears at arthroscopy (positive predictive value (PPV) 61.8%). The negative predictive value of an in-groove LHBT on ultrasound for arthroscopically confirmed subscapularis tears was found to be 78.2%.

The secondary aims of our study were to assess the accuracy of ultrasound in diagnosing LHBT subluxation and to assess the relationship between the LHBT position on ultrasound and subscapularis tears. We investigated the sensitivity of ultrasound in detecting LHBT subluxation and subsequently assessed the correlation between a subluxed LHBT on ultrasound and a confirmed subscapularis tear at arthroscopy.

In our study, ultrasound scanning had poor sensitivity for long heads of biceps subluxation or dislocation (only 50%), with a PPV of 35.3%. Out of 24 patients with a subluxed or dislocated LHBT, only 12 were diagnosed on ultrasound. In keeping with our arthroscopic findings, LHBT subluxation/dislocation on an ultrasound scan was poorly sensitive (22.8%) and a poor predictor of subscapularis tears at arthroscopy (PPV 61.8%).

Eighty-four ultrasound scans were performed by sonographers, whilst 275 were performed by musculoskeletal radiologists. The accuracy in detecting LHBT subluxation was the same in the two groups (90.5%; Table 3). The median time to surgery from an ultrasound scan was 22 weeks (IQR 12-36 (25-75%)).

**Table 3:** Diagnostic accuracy of ultrasound scanning for LHBT subluxation/dislocation LHBT: long head of biceps tendon

	Diagnostic accuracy of ultrasound scanning for LHBT subluxation/dislocation		
	Sonographer	Radiologist	Combined
Actually subluxed/dislocated	3 patients	21 patients	24 patients
Normal/in-groove	81 patients	254 patients	335 patients
True positive	2 patients	10 patients	12 patients
False positive	7 patients	15 patients	22 patients
False negative	1 patient	11 patients	12 patients
True negative	74 patients	239 patients	313 patients
Sensitivity	66.7%	47.6%	50.0%
Specificity	91.4%	94.1%	93.4%
Positive predictive value	22.2%	40.0%	35.3%
Negative predictive value	98.7%	95.6%	96.3%
Accuracy	90.5%	90.5%	90.5%

## Discussion

The primary aim of this study was to assess the anatomical relationship between LHBT position and subscapularis tears at arthroscopy, and our data question the theory that the LHBT subluxes medially in association with subscapularis tears. At arthroscopy, we found a subluxed LHBT was a poor predictor for full-thickness (including upper border) tears with a sensitivity of 17.4%. Furthermore, 82.6% of patients with an arthroscopically confirmed full thickness/upper border tear had a normally located LHBT. The overall correlation between LHBT subluxation and subscapularis tears in our study was weak (R=0.252, p<0.001).

These results contradict the commonly accepted opinion that a subluxed LHBT is associated with a full-thickness subscapularis tear [15]. Given that the majority of patients in our study with a full-thickness subscapularis tear have their LHBT in-groove, it may be that the other anatomical components forming the biceps sling contribute to its stability to a greater extent than previously thought.

It has been previously suggested that the LHBT may sublux due to tears of the transverse humeral ligament, with the subscapularis tendon intact [14]. Godenèche et al. highlighted the importance of the SGHL and CHL to LHBT stability [11]. In their study of patients with confirmed subscapularis tears, 63% of patients had a normal or stretched SGHL/CHL. In those with a normal SGHL/CHL, the LHBT was centrally located 87% of the time, despite having a subscapularis tear. When the SGHL/CHL complex was stretched, the subluxation/dislocation rate of LHBT increased to 68%.

The results from our study seem to support the notion that the subscapularis tendon will often tear without concomitant damage to the SGHL/CHL and therefore without LHB subluxation. It may also be that patients with a subluxed LHBT but no full-thickness subscapularis tear have a stretched or torn SGHL/CHL complex, accounting for the relatively low PPV of a subluxed LHBT for a subscapularis tear in our study (66.7%).

Cadaveric studies have suggested that the CHL plays the most important role in stabilising the LHBT in the intertubercular groove [18]. A histoanatomical study assessing the stabilising sling for the LHBT supports this theory and suggested the subscapularis tendon does not contribute significantly to LHBT stability in the rotator interval, and rather the SGHL and fasciculus obliquus are more important in resisting anterior shearing forces [12].

The prevalence of subscapularis tears in patients undergoing rotator cuff repair in our study was comparable to other studies in the literature. We found that 133/359 (37%) of patients undergoing rotator cuff repair had a subscapularis tendon tear. Narasimhan et al. reported a prevalence of 31.4% [19], whilst other centres have reported a prevalence of between 49% and 59% in patients having arthroscopic rotator cuff repair [4,20-22].

Shi et al. report that LHBT subluxation on MRI has a 97% negative predictive value for arthroscopic full-thickness subscapularis tears [14]. This contrasts with our findings that the LHBT can be in-groove in the presence of subscapularis tears; we found 76 patients at arthroscopy with their LHBT in-groove despite having full-thickness subscapularis tears (negative predictive value 77.3%). In our study, a normally-located LHBT on ultrasound has a negative predictive value of 78.2% for full-thickness subscapularis tears.

Importantly, however, a subluxed LHBT on ultrasound is a poor predictor of and correlates very weakly with subscapularis tears at arthroscopy (R=0.195, p<0.001).

Our study also assessed the accuracy of sonographers and consultant radiologists in detecting LHBT subluxation/dislocation on ultrasound. The majority (77%) of scans were conducted by radiologists; however, the accuracy with which LHBT subluxation/dislocation was diagnosed was similar between the two groups. The limitations of the study include the fact that it is a retrospective study, including data from a single institution. However, the large sample size improves the reliability of the data.

## Conclusions

There is a high prevalence of subscapularis tears in patients undergoing arthroscopic rotator cuff repair (36% in our study). Our data suggest that there is no strong correlation between LHBT subluxation and subscapularis tears, contrary to popular belief and traditional teaching. The authors therefore recommend a high index of suspicion pre-operatively and advise careful arthroscopic assessment for the diagnosis and repair of subscapularis tears, regardless of pre-operative ultrasound findings and the intra-operative position of the LHBT.
